# Convolutional Neural Network Model for Segmentation and Classification of Clear Cell Renal Cell Carcinoma Based on Multiphase CT Images

**DOI:** 10.3390/jimaging9120280

**Published:** 2023-12-14

**Authors:** Vlad-Octavian Bolocan, Mihaela Secareanu, Elena Sava, Cosmin Medar, Loredana Sabina Cornelia Manolescu, Alexandru-Ștefan Cătălin Rașcu, Maria Glencora Costache, George Daniel Radavoi, Robert-Andrei Dobran, Viorel Jinga

**Affiliations:** 1 Department of Fundamental Sciences, Faculty of Midwifery and Nursing, University of Medicine and Pharmacy “Carol Davila”, 050474 Bucharest, Romania; vlad-octavian.bolocan@drd.umfcd.ro (V.-O.B.); cosmin.medar@umfcd.ro (C.M.); maria.costache@umfcd.ro (M.G.C.); 2 Department of Clinical Laboratory of Radiology and Medical Imaging, Clinical Hospital “Prof. Dr. Theodor Burghele”, 050664 Bucharest, Romania; mihaela.secareanu@rez.umfcd.ro (M.S.); elena.sava@rez.umfcd.ro (E.S.); 3 Department of Urology, Clinical Hospital “Prof. Dr. Theodor Burghele”, Faculty of Medicine, University of Medicine and Pharmacy “Carol Davila”, 050474 Bucharest, Romania; stefan.rascu@umfcd.ro (A.-Ș.C.R.); daniel.radavoi@umfcd.ro (G.D.R.); viorel.jinga@umfcd.ro (V.J.); 4 Department of Urology, Clinical Hospital “Prof. Dr. Theodor Burghele”, 050664 Bucharest, Romania; 5 Software Imagination & Vision (Simavi), 013685 Bucharest, Romania; robert.dobran@simavi.ro; 6 Medical Sciences Section, Academy of Romanian Scientists, 050085 Bucharest, Romania

**Keywords:** convolutional neural network, renal cell carcinoma, image segmentation, image classification, artificial intelligence, kidney tumor, European Deep Health toolkit, EDDL, ECVL

## Abstract

(1) Background: Computed tomography (CT) imaging challenges in diagnosing renal cell carcinoma (RCC) include distinguishing malignant from benign tissues and determining the likely subtype. The goal is to show the algorithm’s ability to improve renal cell carcinoma identification and treatment, improving patient outcomes. (2) Methods: This study uses the European Deep-Health toolkit’s Convolutional Neural Network with ECVL, (European Computer Vision Library), and EDDL, (European Distributed Deep Learning Library). Image segmentation utilized U-net architecture and classification with resnet101. The model’s clinical efficiency was assessed utilizing kidney, tumor, Dice score, and renal cell carcinoma categorization quality. (3) Results: The raw dataset contains 457 healthy right kidneys, 456 healthy left kidneys, 76 pathological right kidneys, and 84 pathological left kidneys. Preparing raw data for analysis was crucial to algorithm implementation. Kidney segmentation performance was 0.84, and tumor segmentation mean Dice score was 0.675 for the suggested model. Renal cell carcinoma classification was 0.885 accurate. (4) Conclusion and key findings: The present study focused on analyzing data from both healthy patients and diseased renal patients, with a particular emphasis on data processing. The method achieved a kidney segmentation accuracy of 0.84 and mean Dice scores of 0.675 for tumor segmentation. The system performed well in classifying renal cell carcinoma, achieving an accuracy of 0.885, results which indicates that the technique has the potential to improve the diagnosis of kidney pathology.

## 1. Introduction

Renal cell carcinomas are a group of malignant tumors originating from epithelial cells lining the renal tubules that are divided into multiple histological subtypes. The most common is the clear cell type (70–90%), followed by papillary (10–15%) and chromophobe (3–5%). Other subtypes are collecting duct carcinoma, MiT family translocation renal cell carcinomas, tubulocystic carcinomas, etc. [1].

Among all urogenital types of cancer, renal cell carcinomas are the most prevalent. These malignancies were the sixth most frequently diagnosed cancers in men and the tenth most common cancers diagnosed in women worldwide [2,3]. The majority of renal cell carcinomas are diagnosed incidentally due to the increased availability and usage of imaging modalities, which resulted in a decreasing trend in tumor size and stage [4]. The size and presence of local invasion are key features in the staging and treatment options for renal cell carcinoma (RCC) tumors. When possible, it is desirable to preserve as many nephrons as possible using kidney-sparing surgery for tumor removal. In these cases, cancer recurrence affects 20–40% of patients with localized RCC [5].

Multiphase contrast-enhanced computed tomography (CT) is strongly recommended for diagnosing and staging RCC. Other complementary imaging techniques are magnetic resonance imaging (MRI) and contrast-enhanced ultrasound [6]. Sensitivity for the detection of renal masses using CT is at about 87%, and even higher for lesions greater than 2 cm. Additionally, the specificity in the case of these tumors is 74.5% [7]. The challenging aspects of diagnosing RCC on CT imaging are differentiating malignant tissues from benign tissues (especially clear cell carcinoma versus oncocytoma or fat-free angiomyolipoma), as well as assessing the probable subtype of RCC [8].

It is important to note that this paper brings together the fields of radiology and computer science. Therefore, in order to increase the readability and clarify of the findings, we have added the following list as succinct definitions for key terms we have used:

AI (Artificial Intelligence): AI refers to the development of computer systems that can perform tasks that typically require human intelligence, such as problem solving, learning, and decision making.

Machine Learning: Machine learning is a subset of AI that involves the use of algorithms and statistical models to enable computers to improve their performance on a task without explicit programming, relying on patterns and inference instead.

Convolutional Neural Network (CNN): A type of neural network designed for visual processing, CNNs use convolutional layers to automatically and adaptively learn hierarchical features from data, making them effective for image recognition tasks.

U-net Model: The U-net model is a specific architecture in deep learning, particularly used for image segmentation tasks. It is characterized by a U-shaped architecture, which allows effective feature extraction and segmentation.

RasNet101 Model: RasNet101, or ResNet101, is a specific convolutional neural network (CNN) architecture renowned for its depth, featuring 101 layers. Widely used in image recognition tasks, ResNet architectures, including ResNet101, leverage residual learning to effectively train deep neural network.

EDDL (Embedded Deep Learning): EDDL involves integrating deep learning capabilities into embedded systems, enabling them to perform complex tasks locally without relying on external computing resources.

ECVL (Embedded Computer Vision Library): ECVL refers to a library designed for embedded systems, focusing on computer vision tasks. It aids in implementing computer vision algorithms efficiently on devices with limited computational resources.

Due to recent developments, artificial intelligence now offers an unprecedented opportunity to harness large amounts of data and sophisticated algorithms. It has also been adopted in the medical field, and especially in the radiology domain, as a useful tool for physicians to make more accurate diagnoses and classifications in significantly lower time [9,10]. Whilst the field of artificial intelligence is very wide and there are several artificial intelligence algorithms available, convolutional neural networks (CNNs)—a subclass of deep learning algorithms—have emerged as the most popular modality for processing images [11]. In a survey conducted by Geert Litjens et al. [10], CNNs have been found to be the most preferred approach for medical image interpretation, turning them into current standard practice.

From a hierarchical point of view, deep learning is a subtype of machine learning, which is, in turn, a subclass of artificial intelligence. Deep learning differentiates itself from other machine learning subtypes through the ability of the algorithm to learn, on its own, which features are best for a given computational task, compared to the case in which a human expert chooses certain imaging features that appear to best represent the visual data [12]. Neural networks, the foundation of deep learning algorithms, sometimes known as artificial neural networks (ANNs) or simulated neural networks (SNNs), are used for this purpose. They are known as “neural” because they resemble the way the brain neurons communicate with one another.

**Figure 1:**
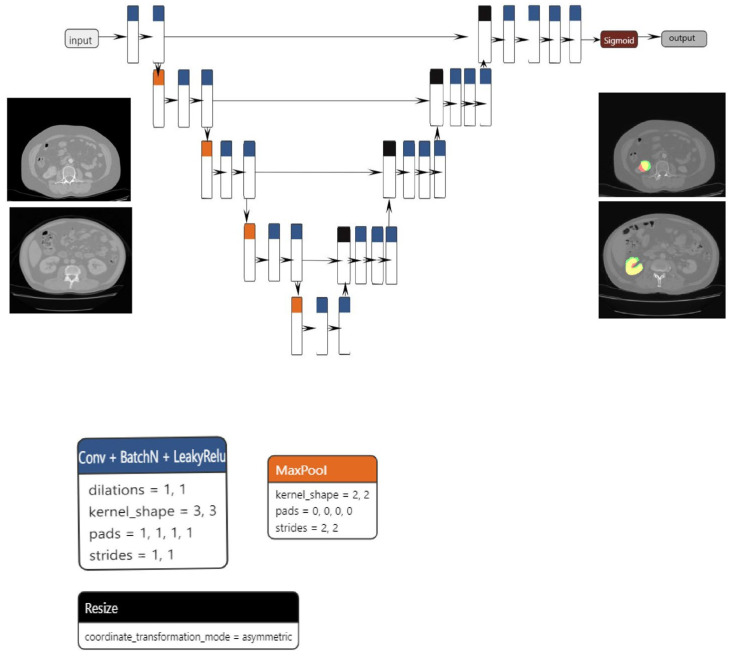
U-net model used.

Neural networks are made up of three main layers—an input layer, a hidden layer (which may comprise several layers), and an output layer (Figure 1). Each node, which links to the next node as an artificial neuron, has a weight and a threshold value. One node is activated and begins sending data to the following layer of the network when its output exceeds the threshold value. When it falls below the threshold, no data are sent. While the input and output layers are always fixed, the hidden layers vary in number, size, and specialization, depending on the type of network.

The primary hidden types of layers combined to build a CNN are the convolutional layer, the pooling layer, and the fully connected layer [13]. The convolutional and the pooling layers act as feature extractors from the base image, while the fully connected layer acts as a classifier. These layers are interconnected in intricate ways, making it challenging to interpret exactly how the network transforms the input into the final output. As a result, it becomes difficult to comprehend which specific features or patterns the CNN focuses on during its analysis, giving CNNs the alias of “black boxes”, due to their underlying complexity [14]. CNNs could extract tangled features from medical images by employing multiple hidden layers of artificial neurons, mimicking the intricate processing of the human visual cortex, with each layer performing specific operations on the input data.

The main areas where CNNs have proven their value in the radiology field are related to object detection, segmentation, and classification [12]. Object detection refers to the ability of the algorithm to determine the presence of objects, as well as their precise location. In medical imaging, it can be used to identify organs, lesions, or tumors. However, the output is represented as a box containing the desired object, as well as other nearby structures. In order to demarcate the exact item though, a segmentation algorithm must be used, as it involves identifying and outlining specific structures or areas of interest within an image, and enables precise measurements, quantification, and analysis of specific regions or abnormalities. Finally, image classification involves categorizing images into predefined classes or categories, distinguishing between different diseases or pathologies based on the visual characteristics present in the image, for example, classifying a CT scan slice as benign or malignant based on the presence of tumor.

The primary metrics for assessing how well the algorithm performs are the Dice score and the Accuracy index [15]. The Dice score is often used to evaluate how well the algorithm can accurately outline or segment an object by measuring the overlap between its predicted segmentation and the actual (ground truth) segmentation. On the other hand, Accuracy is employed to gauge how accurately the algorithm classifies or categorizes different elements.

The significance of the tumor segmentation task is evident via the establishment of the Kidney and Kidney Tumor Segmentation Challenge [16], a competitive event designed to identify the most effective system for the automated semantic segmentation of kidneys and renal tumors. The competition was conducted in the years 2019 and 2021, and the present event is the 2023 iteration. The authors of the study, Zhongchen Zhao et al. [17], were the winners of the 2021 Kidney and Renal Tumor Segmentation Challenge. In their research, they utilized a U-net convolutional neural network (CNN) to perform the segmentation of both the kidney and renal masses. The obtained results showed average Dice scores of 0.908 and 0.860 for the segmentation of the kidney and kidney masses, respectively. Jianhui Wen et al. [18] introduced a squeeze-and-excitation encoder–decoder network called SeResUNet, which bears resemblance to the U-net design. The authors reported somewhat lower Dice scores of 0.672 and 0.545 for the kidney segmentation task and renal tumor segmentation, respectively.

Zheng Gong et al. [19] created a neural network model based on SCNet that can do two things at once: segmenting and classifying kidney tumors. The model achieved a Dice score of 0.846 for tumor segmentation and an accuracy metric of 0.995 for classifying malignant tumors. For sorting kidney tumors into different groups, Alzu’bi et al. [20] suggested making a two-dimensional convolutional neural network with four layers, which they called CNN-4. This model achieved a notable accuracy rate of 92% in effectively discerning between benign and malignant tumors.

Within the framework of this paper, our main aim is to thoroughly assess the effectiveness of a convolutional neural network (CNN) method. This assessment includes two crucial components, segmentation and classification, as they both play a significant role in the diagnosis and management of renal cell carcinoma (RCC). The incorporation of convolutional neural networks (CNNs) in this domain signifies a noteworthy progression in the discipline. Our research strives to conduct a thorough evaluation of the efficacy of CNNs in precisely segmenting and categorizing cases of renal cell carcinoma (RCC). Through the utilization of these performance indicators, our objective is to elucidate the algorithm’s capacities and its potential to augment the diagnosis and treatment of RCC, thereby making a valuable contribution to the enhancement of patient care and outcomes.

## 2. Materials and Methods

The CNN employed in this project is the one developed as part of the European DeepHealth toolkit [21], an open-source framework whose aim is to boost biomedical applications by using cutting-edge deep learning and computer vision algorithms.

The DeepHealth toolkit comprises two integrated software libraries: ECVL (European Computer Vision Library) and EDDL (European Distributed Deep Learning Library), which were created expressly for computer vision and deep learning tasks [22,23,24].

EDDL is a general-purpose deep learning library initially developed to address deep learning requirements in healthcare use cases within the DeepHealth project, and supports widely used deep neural network topologies, including convolutional and sequence-to-sequence models. However, as CNNs have been found to be highly effective [11], with high selectivity and invariance, they are also the most common DNN used for biomedical images. The main layers of CNN are the input layer, convolutional layer, pooling layer, fully connected layers, and the output layer (Figure 1).

The proposed segmentation model, which is based on the U-net architecture, consists of three blocks: convolution, encoding, and decoding. The convolution, encoding, and decoding phases are implemented using convolutional layers, with a stride of one and a total of 23 convolutional layers in the architecture. Following the application of each convolutional layer, batch normalization and LeakyRelu activation are performed. In order to reduce spatial dimensions, the convolutional layers on the encoder block are linked together via max pooling layers.

The decoder block concatenates the resized tensor with the skip connection tensor that was generated by the encoder block that corresponds to it. A sigmoid activation function is subsequently implemented on the output tensor. The dimensions of both the input and output are 1 × 224 × 224. Data augmentation techniques, including elastic transform, color transformation, grid distortion, and image rotation, were applied to both the training and validation sets in order to mitigate the risk of overfitting. The Adam optimizer was utilized to optimize the model.

The primary benefit of the convolutional layer is that it only considers a neuron’s immediate surroundings, and that all neurons inside a layer share the same weights. This significantly decreases the number of parameters and, hence, the memory space needed to store a layer of this type. Additionally, pooling is an operation that is used to reduce the scale of the input. Pooling takes subsamples of the convolutional layer to feed the next layer, acting as a powerful detector of patterns independently of their relative position in the image. Weight sharing in convolutional layers combined with pooling schemes (max or average pooling) allows the extraction of position-invariant relevant properties.

For the current project, the main use case was image classification and segmentation in the process of diagnosing renal tumors. As a result, three main tasks were defined: kidney segmentation, tumor segmentation, and tumor classification.

Regarding medical image segmentation tasks, well-known architectures that are supported by the EDDL library are U-net and V-net. U-net was first introduced in 2015 and has shown very good performance on very different biomedical segmentation applications [25] and was the architecture used for both kidney and tumor segmentation tasks.

**Figure 2:**
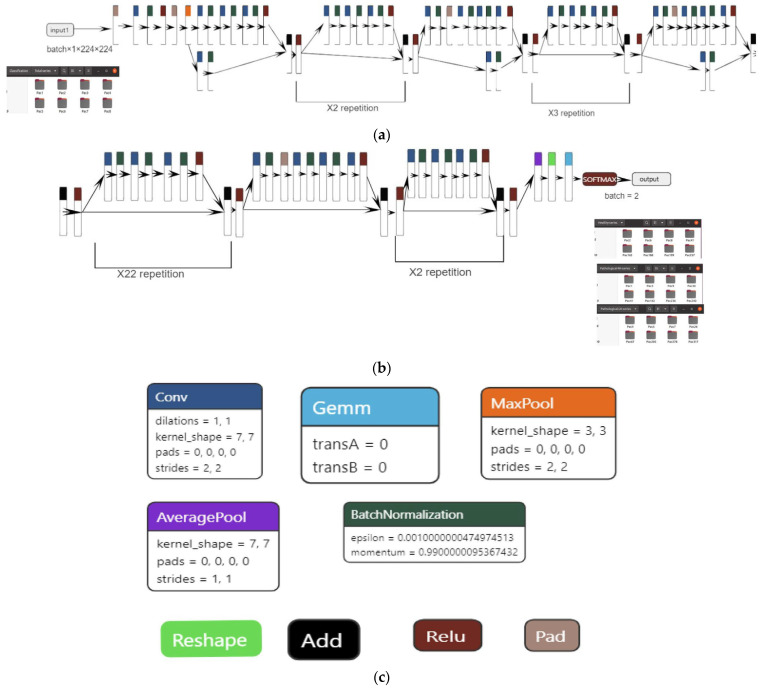
(**a**)—ResNet101 part 1. (**b**)—ResNet101 part 2. (**c**)—ResNet101 part 3.

For the classification task, the model used was based on the resnet101 architecture (Figure 2a–c), which, as the name suggests, has 101 layers and can classify up to 1000 classes. However, for this paper, we restricted the use to up to two classes. The classification model is built upon the ResNet101 architecture, which includes 33 residual blocks and a total of 104 convolutional layers. The first layer has a kernel size of 7 × 7 and a stride of 2, while the subsequent levels have kernel sizes of 1 × 1 and 3 × 3, all with a stride of 1. Each convolutional layer is followed by a batch normalization step. To ensure the initial collection of the most significant features, the network employs max pooling at an early stage. Conversely, average pooling is employed towards the end to accentuate the overall content of the feature maps. The network utilizes the Relu activation function throughout all layers, although in the final step of the architecture, the softmax function is applied. The final layer of the network employs a matrix multiplication operation, specifically implemented as General Matrix Multiply (GEMM).

ECVL is designed to serve two main purposes, allowing an easy integration and data exchange between existing libraries, including EDDL, and the availability of performance testing frameworks, which will allow repeatable experiments on large scale datasets to verify the impact of different modifications.

The image class represents the core of the entire ECVL library. It is an object that stores data (either images/videos or raw data) in a multi-dimensional dense numerical single or multi-channel tensor. The tensor is a vector or matrix used in machine learning for storing data. The ECVL library supports all common data formats, as well as NIfTI and DICOM, providing both reading, writing, and most of the image manipulation and processing functionalities. Furthermore, a visualizer for 3D volumes, such as CT scans, allows one to observe different slices of a volume from different views.

Data partitioning is a key step for training neural networks. The split process is performed via ECVL in order to provide EDDL with the required data for training and validation steps. The data augmentation process allows the artificial enlargement of a dataset by perturbing the training data in realistic ways, mimicking variability between different CT scanners or image acquisition timing to prevent overfitting and thus improving the training process of neural networks and increasing the final accuracy. Overfitting is an undesirable machine learning behavior that occurs when the machine learning model gives accurate predictions for training data but not for test data [26]. The ECVL library includes all the augmentation strategies commonly exploited in the literature, such as flipping, adding salt-and-pepper noise, blurring an image, or adjusting the contrast [27].

The cooperation between EDDL and ECVL was achieved by defining the DeepHealth Dataset Format (DDF), which is based on the YAML syntax. The YAML is a data serialization language mainly used for writing configuration files. Serialization is a process where one application or service that has different data structures and is written in a different set of technologies, and can transfer data to another application using a standard format. This format defines all the information such as the name and description of the dataset, its classes and features, a list of image or volume paths, and a split indicating how to divide images into training, validation, and test sets. The DeepHealth Dataset Format also allows the specification of segmentation masks for each input entry. An ECVL module is provided to parse and load DDF defined datasets into the specific dataset class. The library’s interface is based on two main functions that convert ECVL Image(s) into EDDL Tensor(s) and vice versa.

The dataset provided was used both for training the model in kidney segmentation and RCC classification and for performing inferences. The raw dataset consisted of DICOM images corresponding to four-phase contrast-enhanced CT (pre-contrast, arterial, venous, and excretory phase). For this project, only arterial phase images were used. After installing and configuring all necessary tools, the data have been anonymized and annotated. In the case of segmentation, a segmentation of the area of interest was performed, which was then saved in NIfTI format. For the classification task, each patient was labeled with or without a tumor. Then, the relevant series were extracted from the raw DICOM data. Using a tool to create YMLs and selecting the required parameters, the data were transformed into DeepHealth Dataset Format. Afterwards, the model was trained on this dataset; inferences were performed in order to determine whether the resulting model was suitable; the final step was validating the results.

These steps were performed for both the segmentation and classification of the right and left kidney, separately. Tumor segmentation was executed on the right and left kidney.

We used the following parameters for segmentation and classification, Table 1.

The number of batches between synchronizations of weights is a crucial configuration parameter to achieve speedups close to the optimum. This parameter is dynamically adjusted to bound the communications overhead to be lower than the percentage given as a reference. Another important parameter is the batch size. While all the tensors necessary to conduct all the computations of the train batch operation fit in the memory of the GPU, larger batch sizes allow the EDDL to better leverage the full potential of GPUs.

The raw dataset consisted of 457 kidneys from patients with a healthy right kidney, 456 kidneys from patients with a healthy left kidney, 76 kidneys from patients with a pathological right kidney, and 84 kidneys from patients who had a pathological left kidney, Table 2 and Table 3.

The training process begins with an input YAML file, which serves as a mapping tool. This YAML file specifies how to handle the dataset, DICOM data, and NIfTI data, especially in cases involving segmentations. On the other side of the training pipeline, the output is comprised of two primary components: a trained model saved in the ONNX format, which is specifically designed for representing machine learning models, and a collection of PNG images.

These PNG images are generated based on the initial DICOM data and serve different purposes depending on the type of training being performed. For segmentation tasks, some PNG images display the results of the model’s predictions, showcasing the regions it has identified, while others show the ground truth masks for comparison. In classification tasks, the PNG images are organized into separate folders based on the predicted classes assigned by the model. This output structure helps to assess the performance and effectiveness of the trained model. Some of the factors relevant in deciding whether the model is suitable or not are key performance indicators (KPIs), as seen in Table 4. KPIs are data points and measurement tools that can be used to monitor and evaluate the quality of services provided by a radiology operation [28].

Apart from the time-to-model-in-production, time-of-training-models, and time-of-pre-processing-images KPIs (considering that these time-consuming tasks have to be carried out only once at the beginning of the project), all the other indicators can and will be used in this paper to assess the efficiency of the CNN model in clinical practice.

The study was peer-validated and approved by the Ethics Committee of the “Profesor Dr. Th. Burghele” Clinical Hospital, Bucharest, Romania (approval number 2/2021), and all procedures in the study respected the ethical standards of the Helsinki Declaration. Informed consent was obtained from all participants.

## 3. Results

### 3.1. Model Comparison

U-net was chosen above SegNet and NablaNet for segmentation, Table 5 and Table 6. Before library training, datasets were augmented. At the time the SegNet libraries did not handle DICOM data; therefore, we transformed DICOM and NIfTI data to PNG, which was unsatisfactory. The neural network training of the NablaNet model using only NIFTI data did not enhance Dice scores. Our U-net model yielded the best results, achieving a Dice score of up to 0.81. We used ResNet101 and ResNet152 for the classification task and found that ResNet101 worked best.

The following tables further illustrate the comparison with other models, i.e., ResNet101, that we have tested before settling on U-net.

### 3.2. Pre-Processing

The pre-processing of data, as seen in Table 7, consisted mainly of time series epoching/segmentation, filtering, artefact detection/rejection; the areas of interest were highlighted and the radiological description was added. Anonymization steps were first performed. Afterwards, depending on the case (segmentation vs. classification task), a segmentation of the area of interest is performed, or each patient was labelled as having a tumor or not. Afterwards, the relevant series containing the needed information from each patient were extracted.

The time to run each step was as follows:

- 5 min/patient to anonymize;
- 12 min/patient to perform the segmentation;
- 1 min/patient series extraction time;
- 12 h to develop and 30 min to install the YML tool based on DICOM and NIfTI data; This was carried out only once and does not depend on the task or the organ;
- 2 min/patient to run the pipeline time.

### 3.3. KPIs Results

As stated in Section 2, the main KPIs used to assess the efficiency of the model in clinical practice are time-to-diagnose, kidney segmentation quality, tumor segmentation quality, and classification quality of renal cell carcinoma (RCC). In the following sections, all these KPIs are addressed.

#### 3.3.1. Kidney and Tumor Segmentation Quality

Kidney segmentation quality measures how well the model predicts the zone of interest using the Dice score as indicator, as seen in Table 8. This Dice coefficient measures the overlap between the two NIfTI masks: first the original segmentation mask with the kidney and the second mask is the one predicted by the model. The target Dice score for the project was 0.8. The results are presented in Table 8.

**Figure 3:**
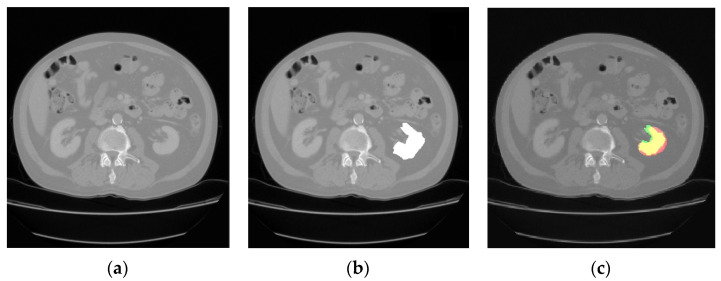
CT image of a healthy kidney (**a**); the medical expert segmentation (shown in white) (**b**); and the result of the model’s prediction (**c**).

**Figure 4:**
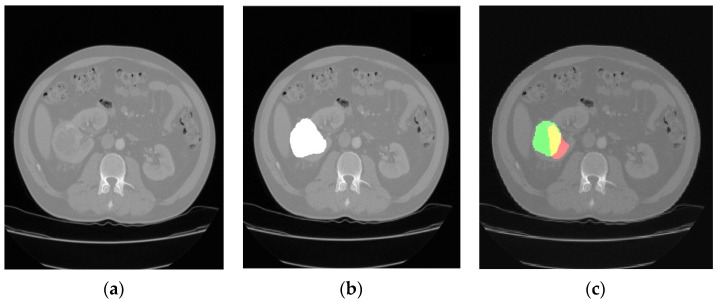
CT image of a kidney with clear cell renal cell carcinoma (**a**); the medical expert tumor segmentation (shown in white) (**b**); and the result of the tumor location prediction (**c**).

The comparison of the output results of the segmentation network and the expert segmentation of a healthy kidney is shown in Figure 3, while for the pathological kidney (renal tumor), the comparison of the output results of the segmentation network and the expert segmentation of a healthy kidney is shown in Figure 4. Regarding the potential bias of the medical experts, although it cannot be eliminated, we have striven to greatly reduce this bias by triple checking the segmentation of the area of interest. Therefore, a radiology resident (V.O.B.), a radiology fellow (G.M.C.), and a radiology attending physician (C.M.), with a combined 25 years of experience imaging renal cell carcinoma, utilized 3D Slicer [29] to manually segment the dataset. Concerning the color scheme utilized below, the red area represents the prediction, the green area the ground truth. The yellow area, therefore, represents the correctly predicted pixels.

#### 3.3.2. Classification Quality of Clear Renal Cell Carcinoma (cRCC)

The dataset is classified by the trained model in one of two classes: patient with normal kidney or patient with tumor, Table 9. The validity of the model was assessed by using the accuracy metric. The patient was diagnosed based on the number of images belonging to them that were classified as healthy or containing a tumor. The overall score was calculated based on how many patients were diagnosed correctly.

The complete dataset was split into 70-20-10 training, validation, and test datasets (occasionally 70-10-20). Validation and metric calculation followed each epoch (dice for segmentation, accuracy for classification). If the metric for that epoch exceeded the best prior one, an ONNX model was recorded. Finally, the model from the epoch with the best validation dataset performance was used. Testing this ONNX model on the test dataset was the second stage. A C++ inference source code file was created to display the prediction on the test dataset and compute the ONNX model’s performance. A C++ source code file that displays the projection but does not compare it to the ground truth was also created.

Segmentation and classification validation and testing differ. Each image’s Dice metric is calculated during validation and testing for segmentation. Image measurements are averaged to determine the total metric. Two categorization methods are utilized. In the first method, accuracy is calculated for each image and averaged throughout the validation or test dataset. The total measure shows how successfully the model diagnosed each image. In the second technique, accuracy metrics are determined for each image and a patient’s diagnosis is based on whether more of their images are healthy or not. How many cases were accurately diagnosed determines the final score.

To prevent overfitting, the dataset was split into three: training, validation, and testing. Even if the process ran for 100 epochs, only trained models that performed well on the validation dataset (that was not used in the training proper) were considered. Further testing was conducted on the test dataset to check whether the trained model could perform on other input data. Thus, the method of early stopping was used to prevent overfitting. Additionally, a diverse and large dataset was also used to prevent overfitting, as well as data augmentation. We implemented various data augmentation techniques in our model to mitigate the risk of overfitting. These techniques included resizing to dims with cubic interpolation, conversion to 32-bit float, mirroring with probability, rotation by angle, addition of Poisson noise, Gamma contrast, Gaussian blur, and Coarse dropout.

#### 3.3.3. Time-to-Diagnose

The time-to-diagnose KPI measures how much time it takes to diagnose a renal clear cell carcinoma for a given patient, Table 10. The baseline value indicates the time that a diagnosis of a clear renal cell carcinoma would take without using machine learning tools, while the final stage indicates the time to diagnose the tumor using the libraries and the trained model.

## 4. Discussion

In this study, the data preprocessing was of the uttermost importance, as it prepared the input for the subsequent analysis. The process encompassed several essential procedures, namely time series epoching/segmentation, filtering, artifact detection/rejection, and the subsequent identification of regions of interest, which were then subjected to radiological description. The first stage included in the process was anonymization, which aimed to safeguard patient privacy and ensure the security of the data. Various methods were conducted depending on the specific goal at hand, such as segmentation or classification. In the context of segmentation tasks, the region of interest underwent a process of segmentation, whereas in classification tasks, patients were assigned labels indicating the presence or absence of a tumor. Following this, pertinent data sets comprising crucial patient information were extracted. The average duration of the anonymization method each patient was 5 min, while the segmentation technique took around 12 min per patient. Additionally, the series extraction procedure required approximately 1 min per patient. In addition, the YML tool was developed and implemented using DICOM and NIfTI data, requiring a total of 12 h. It is important to note that this was a singular undertaking and not contingent on the particular activity or organ involved. Ultimately, the execution of the pipeline for each individual patient necessitated a time commitment of 2 min. The pre-processing stages established the groundwork for the later evaluation of key performance indicators (KPIs).

The assessment of the model’s effectiveness in clinical practice involved the consideration of various key performance indicators (KPIs), as detailed in Section 2. The key performance indicators (KPIs) encompassed in this study are time-to-diagnose, kidney segmentation quality, tumor segmentation quality, and classification quality of renal cell carcinoma (RCC). A thorough analysis was conducted on each of these key performance indicators (KPIs) to gain a comprehensive understanding of the model’s performance.

A score called the Dice score was used to check the quality of the kidney segmentation. This score measures how much the predicted segmentation mask made by the model and the original segmentation mask for the kidney overlap. A benchmark Dice score of 0.8 was set as the aim for the project, representing the ideal level of accuracy in kidney segmentation. The evaluation of the classification accuracy of clear renal cell carcinoma (cRCC) was conducted by assessing the model’s capacity to correctly categorize patients into two categories: those with a healthy kidney and those with a tumor. The metric employed to assess the validity of the model was accuracy. The diagnosis of patients was determined by assessing the number of images identified as either healthy or containing a tumor. Subsequently, an overall accuracy score was computed to evaluate the correctness of patient diagnoses.

The accurate segmentation of tumors holds a critical position in the diagnostic and therapeutic processes related to clear cell renal cell carcinoma (cRCC). The precise delineation of the tumor mass is crucial for effective surgical planning, especially in cases where partial nephrectomy is considered. Partial nephrectomy involves the selective removal of only the malignant tissue, preserving the surrounding healthy kidney tissue. Therefore, it becomes paramount to accurately identify and outline the tumor boundaries in relation to the renal collective system for optimal medical decision-making.

The model we have suggested demonstrated a segmentation performance of 0.84 for the task of kidney segmentation, accompanied by a mean Dice score of 0.675 for tumor segmentation. The aforementioned values exhibit a slight decrease in comparison to the results reported by Zhongchen Zhao et al. [17], which were 0.908 and 0.860, respectively. In contrast, the Dice scores for both kidney segmentation and kidney mass segmentation, which were 0.84 and 0.675, respectively, exhibited superior performance compared to the metrics provided by Jianhui Wen et al. [18]. Their scores were 0.672 for kidney segmentation and 0.545 for kidney mass segmentation.

Our model achieved a segmentation performance score of 0.675 for renal masses, which was a little lower than the 0.846 score reported by Zheng Gong et al. [19]. However, it is important to note that the accuracy measure was more consistent across the studies. Our model demonstrated an accuracy score of 0.885, whereas Zheng Gong et al. reported an accuracy measure of 0.995. Additionally, there was a slighter difference in the accuracy measure when compared to the model that Dalia Alzu’bi et al. [20] suggested. Their model achieved an accuracy rate of 0.92, whereas our model earned a score of 0.885.

The metric known as “time-to-diagnose” functioned as a crucial key performance indicator (KPI) that measured the duration necessary to diagnose renal clear cell carcinoma in individual patients. The baseline value denoted the duration required for tumor diagnosis without the utilization of machine learning techniques, whereas the final stage indicated the time taken to diagnose the tumor with the aid of libraries and the trained model. The key performance indicators (KPIs) in question offer a comprehensive evaluation of the model’s performance within the context of clinical practice. They provide insights into the model’s efficiency and efficacy in facilitating the diagnosis of renal cell carcinoma.

### Limitations of the Study

The findings of this study necessitate careful consideration within the scope of certain limitations. Firstly, the size of our patient cohort, in contrast to the datasets commonly available for artificial intelligence (AI) segmentation challenges, was comparatively smaller. This limitation stemmed from the labor-intensive nature of manual data preprocessing, impacting the breadth and diversity of the dataset. Consequently, the accuracy of our model may be tempered when benchmarked against what might have been achievable with a more extensive and diverse dataset. This constraint highlights the intrinsic trade-off between dataset size and the rigor of manual preprocessing, influencing the model’s performance metrics.

Secondly, the constrained dataset introduces the potential for overfitting, particularly attributable to the inclusion of skip connections and additional layers in the expanding path. These architectural choices resulted in a notable escalation in the number of parameters, potentially making the model more prone to fitting noise in the training data rather than capturing essential patterns. The interplay between dataset limitations and model complexity underscores the delicate balance required in designing neural network architectures for optimal performance.

Furthermore, our study explored specific segmentation and classification architectures, namely U-net, SegNet, NablaNet, ResNet101, and ResNet152. While U-net demonstrated superior performance among the segmentation models and ResNet101 and ResNet152 were the sole models considered for classification, other architectures such as EfficientNet for segmentation and V-Net and SCNet for both tasks could have yielded promising results. However, due to resource constraints and an extended testing period, our study was circumscribed in its exploration of alternative architectures. This underscores the importance of acknowledging the limitations in the range of models tested, highlighting potential avenues for future research to explore a more comprehensive spectrum of neural network architectures.

Lastly, yet another aspect that required careful consideration was the possible bias of the human specialists performing the segmentation of the areas of interest. In this regard, we have devised a system meant to minimize the potential issues: three individuals—comprising a radiology resident (V.O.B.), a radiology fellow (G.M.C.), and a radiology attending physician (C.M.)—leveraged their cumulative 25 years of experience in renal cell carcinoma imaging to manually segment the dataset using 3D Slicer [29].

## 5. Conclusions

This study concludes that data pre-processing is essential for key performance indicator analysis. With meticulous care, time series segmentation, filtering, artefact detection, and region identification were performed during pre-processing. Initial anonymization aimed to safeguard patient privacy and data security. Regardless of the goal, various methods were used to partition regions and mark patients for tumor identification. Developing and using the DICOM- and NIfTI-based YML tool required a large initial investment. Its necessity in improving data analysis and model evaluation has been shown. KPIs provided a detailed assessment of the model’s clinical efficacy. The model tried to achieve a Dice score of 0.8 to segment the kidneys precisely, proving its precision. Additionally, the model’s precision in categorizing patients with clear renal cell carcinoma (cRCC) was assessed, focusing on accuracy. The KPI “time-to-diagnose” highlighted the efficiency advantages that machine learning can bring to renal clear cell carcinoma diagnosis. In conclusion, these key KPIs demonstrate the model’s clinical efficacy in renal cell cancer diagnosis. A thorough review of key performance indicators (KPIs) and suitable data modification procedures before analysis are crucial to determining how effectively a model works and how it can improve radiology and oncology patient care. In light of the above, this study highlights the vital role of careful data preparation in assessing a model’s effectiveness in diagnosing kidney cancer. The findings emphasize the importance of using specific measures, such as a Dice score of 0.8 and efficient diagnosis time, to gauge and enhance the model’s performance for improved patient care.

## Figures and Tables

**Table 1:** Parameters.

Segmentation	Classification
Learning rate: 0.0001	Learning rate: 0.0001
Epochs: 100	Epochs: 100
Batch size: 8	Batch size: 20
Loss: DICE	Loss Binary Cross Entropy
Metric: DICE	Metric: accuracy
Height: 224	Height: 224
Width: 224	Width: 224

**Table 2:** Raw dataset of healthy kidneys.

Dataset	Number of Patients	Size Original Data (GB)	Size Processed Data (GB)	Size Segmentations (GB)	Number of Images
Healthy right kidney	457	345.5	59.22	62.3	119,853
Healthy left kidney	456	344.5	59.22	62.2	119,529

**Table 3:** Raw dataset of pathological kidneys.

Dataset	Number of Patients	Size Original Data (GB)	Size Processed Data (GB)	Size Segmentations (GB)	Number of Images
Pathological right kidney	76	31.65	7.48	7.43	13,248
Pathological left kidney	84	35.93	8.76	9.32	18,133

**Table 4:** The main KPIs that were calculated throughout the entire project.

Time-to-diagnose	measures how much time it takes a medical professional to diagnose urology problems for one patient
Time-to-model-in-production ttmip	measures the time necessary to complete an entire training session with sufficient epochs to assure convergence to the desired performance on the test set (epochs defines the number times that the learning algorithm will work through the entire training dataset)
Time-of-training-models totm	measures the execution time of the training process per epoch
Time-of-pre-processing-images toppi	measures the time necessary to accomplish all the preprocessing tasks (installing software, anonymizing and annotating data, etc.)
Kidney segmentation quality	measures how well the model predicts the region of interest using the DICE score as indicator
Tumor segmentation quality	measures how well the model predicts the region of interest using the DICE score as indicator
Classification quality of renal cell carcinoma (RCC)	The metric used for this KPI is accuracy

**Table 5:** Segmentation model comparison.

Model	Dice Score Value
SegNet	0.025
NablaNet	0.43
U-net	0.81

**Table 6:** Classification model comparison.

Model	Accuracy Score Value
ResNet152	0.71
ResNet101	0.82

**Table 7:** Pre-processing of data.

	Task	Organ	Anonymization + Segmentation Time	Total Time in Intermediate Stage	Total Time in Final Stage
Time of pre- processing images	Segmentation	Right kidney	17 min/patient	20 min/patient + 12.5 h fixed time	20 min/patient + 12.5 h fixed time
		Left kidney	17 min/patient	20 min/patient + 12.5 h fixed time	20 min/patient + 12.5 h fixed time
	Classification	Right kidney	5 min/patient	5 min/patient + 12.5 h fixed time	5 min/patient + 12.5 h fixed time
		Left kidney	5 min/patient	5 min/patient + 12.5 h fixed time	5 min/patient + 12.5 h fixed time

**Table 8:** Results of kidney and tumor segmentation quality.

	Task	Organ	Expected Target at the End of the Project	Intermediate Stage	Final Stage
Segmentation	Kidney segmentation quality (Dice score)	Right kidney	0.80	0.81	0.84
		Left kidney	0.80	0.80	0.84
	Pathological segmentation quality (dice score)	Pathological right kidney	0.80	0.73	0.71
		Pathological left kidney	0.80	0.73	0.64

**Table 9:** Dataset for classification quality of clear renal cell carcinoma.

	Task	Organ	Expected Target at the End of the Project	Intermediate Stage	Final Stage
Classification	Classification quality of clear renal cell carcinoma for patients (accuracy)	Right kidney	0.70	0.94	0.92
		Left kidney	0.70	0.85	0.85

**Table 10:** Time-to-diagnose KPI.

	Baseline Stage	Final Stage
Time-to-diagnose	420 s/patient	12 s/patient

## Data Availability

The data are not publicly available due to the contract of grant agreement No. 825111.
