# Correction: Bolocan et al. Convolutional Neural Network Model for Segmentation and Classification of Clear Cell Renal Cell Carcinoma Based on Multiphase CT Images. *J. Imaging* 2023, *9*, 280

**DOI:** 10.3390/jimaging10020035

**Published:** 2024-01-29

**Authors:** Vlad-Octavian Bolocan, Mihaela Secareanu, Elena Sava, Cosmin Medar, Loredana Sabina Cornelia Manolescu, Alexandru-Ștefan Cătălin Rașcu, Maria Glencora Costache, George Daniel Radavoi, Robert-Andrei Dobran, Viorel Jinga

**Affiliations:** 1 Department of Fundamental Sciences, Faculty of Midwifery and Nursing, University of Medicine and Pharmacy “Carol Davila”, 050474 Bucharest, Romania; vlad-octavian.bolocan@drd.umfcd.ro (V.-O.B.); cosmin.medar@umfcd.ro (C.M.); maria.costache@umfcd.ro (M.G.C.); 2 Department of Clinical Laboratory of Radiology and Medical Imaging, Clinical Hospital “Prof. Dr. Theodor Burghele”, 050664 Bucharest, Romania; mihaela.secareanu@rez.umfcd.ro (M.S.); elena.sava@rez.umfcd.ro (E.S.); 3 Department of Urology, Clinical Hospital “Prof. Dr. Theodor Burghele”, Faculty of Medicine, University of Medicine and Pharmacy “Carol Davila”, 050474 Bucharest, Romania; stefan.rascu@umfcd.ro (A.-Ș.C.R.); daniel.radavoi@umfcd.ro (G.D.R.); viorel.jinga@umfcd.ro (V.J.); 4 Department of Urology, Clinical Hospital “Prof. Dr. Theodor Burghele”, 050664 Bucharest, Romania; 5 Software Imagination & Vision (Simavi), 013685 Bucharest, Romania; robert.dobran@simavi.ro; 6 Medical Sciences Section, Academy of Romanian Scientists, 050085 Bucharest, Romania

In the original publication [1], references “[30–37]” were cited by mistake. The citations have now been deleted in the References section.

In the last paragraph of the Discussion, the citation should be updated as follows: 

“Lastly, yet another aspect that required careful consideration was the possible bias of the human specialists performing the segmentation of the areas of interest. In this regard, we have devised a system meant to minimize the potential issues: three individuals—comprising a radiology resident (V.O.B.), a radiology fellow (G.M.C.), and a radiology attending physician (C.M.)—leveraged their cumulative 25 years of experience in renal cell carcinoma imaging to manually segment the dataset using 3D Slicer [29]”.

The authors state that the scientific conclusions are unaffected. This correction was approved by the Academic Editor. The original publication has also been updated.
